# Single‐cell profiling reveals novel cellular heterogeneity of monocytes during Hymenoptera venom allergy

**DOI:** 10.1002/clt2.12151

**Published:** 2022-05-05

**Authors:** Wen‐Cheng Chao, Wen‐Ting Liao, Jing‐Rong Wang, Hsiao‐Ni Sung, Hsin‐Hua Chen, Fang‐Ping Lin, Jou‐Yu Huang, Kuan‐Ting Liu, Tai‐Ming Ko

**Affiliations:** ^1^ Department of Critical Care Medicine Taichung Veterans General Hospital Taichung Taiwan; ^2^ Department of Biological Science and Technology National Yang Ming Chiao Tung University Hsinchu Taiwan; ^3^ Division of Allergy, Immunology and Rheumatology Department of Internal Medicine Taichung Veterans General Hospital Taichung Taiwan; ^4^ School of Medicine National Yang Ming Chiao Tung University Taipei Taiwan; ^5^ Institute of Bioinformatics and Systems Biology National Chiao Tung University Hsinchu Taiwan; ^6^ Institute of Biomedical Sciences Academia Sinica Taipei Taiwan; ^7^ Center for Intelligent Drug Systems and Smart Bio‐devices (IDS^2^B) National Yang Ming Chiao Tung University Hsinchu Taiwan

**Keywords:** immunology, immunotherapy, insect hypersensitivity, molecular allergy

## Abstract

**Background:**

Hymenoptera stings can induce dysregulated inflammation and immediate hypersensitivity reactions including anaphylaxis. However, the molecular mechanisms underlying peripheral immune responses during Hymenoptera venom allergy (HVA) remain elusive.

**Methods:**

Here we determined the single‐cell transcriptomic profiling from highly heterogeneous peripheral blood cells in patients with HVA through unbiased single‐cell RNA sequencing and multiple models of computational analyses.

**Results:**

Through clustering analysis by uniform manifold approximation and projection, we revealed an increased number of monocytes in the acute phase and identified innate immune responses, leukocyte activation, and cellular detoxification as the main involved biological processes. We used filter analysis to identify that CLU that encodes clusterin was highly expressed in monocytes, and the co‐expressed genes of CLU further supported the key role of monocyte. We further used pseudo‐temporal ordering of cells and scRNA velocity analysis to delineate disease‐associated monocyte lineages and states in patients with HVA.

**Conclusions:**

Our comprehensive molecular profiling of blood samples from patients with HVA revealed previously unknown molecular changes, providing important insights into the mechanism of venom allergy and potential therapeutic targets.

## 1 TO THE EDITOR

The immune response induced by Hymenoptera stings can cause dysregulated inflammation and systemic allergic responses.^1^ A wide range of clinical manifestations including anaphylactic shock result from Hymenoptera venom allergy (HVA).^2^ In Europe, prevalence of systemic anaphylactic sting reactions ranges from 0.3% to 7.5%.^3^ The current emergency treatment can decelerate the development of systemic allergic reactions by injecting non‐specific immune regulators, such as corticosteroids.^4^ However, even if the pathogenic mechanism is inferred from previous observations, molecular mechanisms underlying peripheral immune responses during HVA are elusive, which is mainly because few genomics datasets are available for understanding the immune response caused by *Vespa* stings.

Here we determined the blood single‐cell signatures from highly heterogeneous peripheral blood cells in patients with HVA through unbiased single‐cell RNA sequencing and multiple models of computational analyses.

To investigate how the molecular and cellular profiles of blood leukocytes are altered in patients with HVA compared to those in healthy control (HC) individuals, we performed single‐cell transcriptome analysis of 11 blood leukocyte samples (*n* = 7892 cells) by single‐cell RNA sequencing (scRNA‐seq). Four HVA blood samples (*n* = 2056 cells) were obtained from two HVA patients (P1, *n* = 1503 cells; P2, *n* = 553 cells), one sample each at acute and recovery stages. Seven HC blood samples (*n* = 5836 cells) were obtained from seven HC individuals.

**FIGURE 1:**
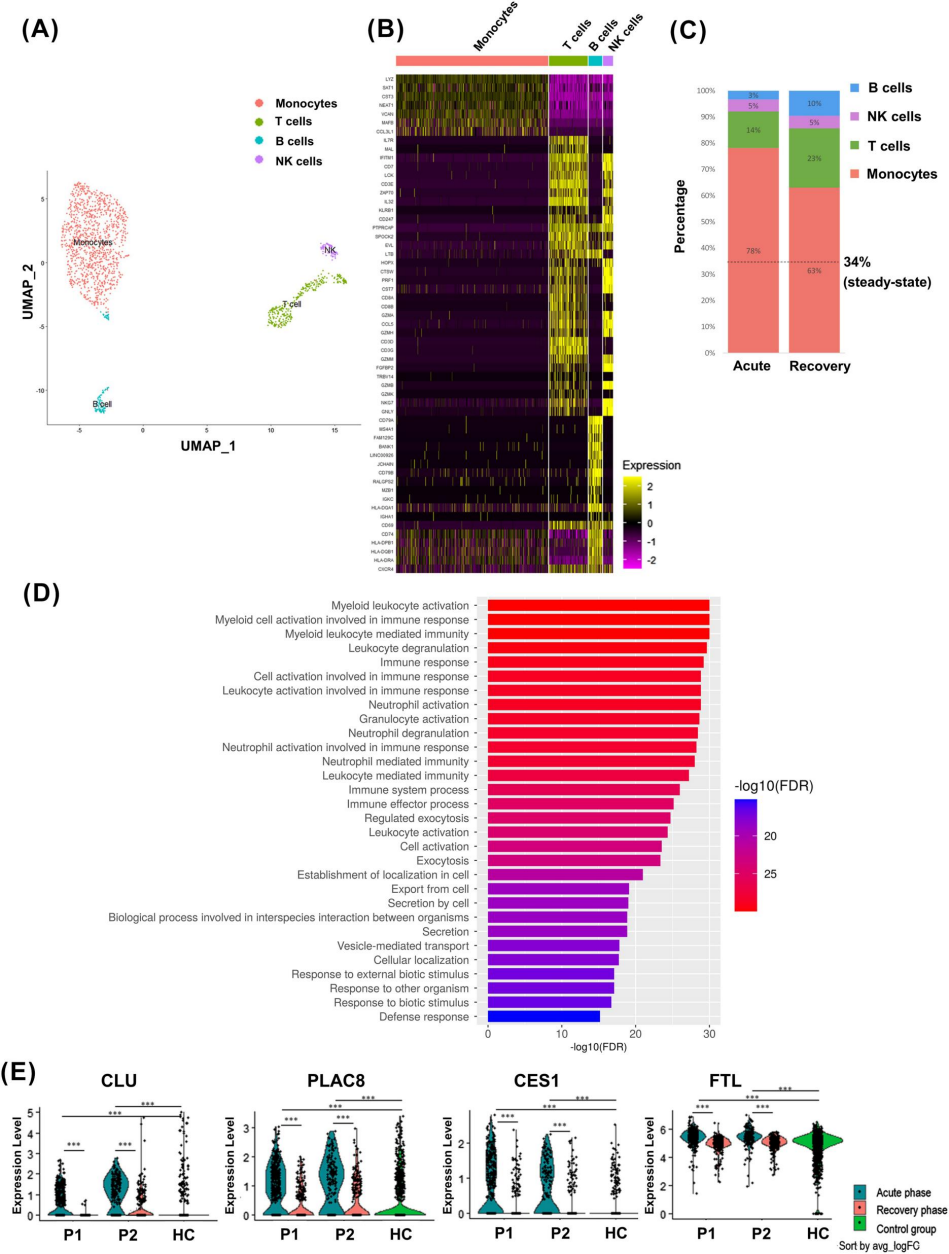
Single‐cell profiling of peripheral blood mononuclear cells (PBMCs) in patients with Hymenoptera venom allergy (HVA). (A) PBMCs were analyzed from patients with HVA and visualized with uniform manifold approximation and projection (UMAP). (B) Gene‐expression heatmap of the marker genes corresponding to each cluster are identified. Genes are represented in rows and cell clusters in columns. Marker genes are indicated to the left. (C) The frequency of each cell type is depicted in the columns. The steady‐state condition was determined by the frequencies of monocytes in HC individuals. (D) Enriched gene ontology (GO) functions of regulated genes involved in HVA is presented by a histogram by using the differentially expressed genes (DEGs) shared in two acute HVA patients. (E) Differentially expressed gene in monocyte cell population were determined in each patient with HVA. The differentially expressed genes were identified by Wilcoxon Rank Sum test and *p* value were corrected by Bonferroni correction using all genes in the dataset. (avg_log2FC: log fold‐change of the average expression)

To establish a baseline profile of the cell populations, we performed an initial unbiased uniform manifold approximation and projection (UMAP) clustering using all 11 samples (Figure 1A). This analysis generated four major cell clusters that were subsequently categorized into the following four major cell types according to their individual transcriptome profiles and previously reported cell‐type markers: monocytes (*LYZ*), T cells (*CD3E*), B cells (*CD79 A*), and NK cells (*KLRF1*; Figure 1B). To elucidate the immunological changes in HVA patients, we investigated the relative proportions of immune cells in peripheral blood mononuclear cells (PBMCs) in the acute stage compared with those in the recovery stage. We found that monocytes encompassed the major cell‐populations in the acute phase (Figure 1C).

Recruitment of monocytes to the site of inflammation is critical for host defense.^5^ Since HVA is accompanied by a strong inflammatory response in multiple sites of inflammation, frequency of monocytes was potentially increased via monocyte activation and recruitment mechanisms. Furthermore, previous findings also supported the roles of venom in the induction of monocyte activation.^6^

To characterize the global transcriptomic differences in monocytes between acute HVA patients and HC individuals, we performed gene ontology (GO) enrichment analyses that identified the main underlying biological processes including leukocyte activation and myeloid leukocyte activation in HVA (Figure 1D).

To further identify critical transcriptomic biosignatures in monocyte cell clusters involved in HVA, we performed feature selection using multiple filters from the shared differentially expressed genes (DEGs) pools in monocytes. These filters were designed to identify DEGs with the three major properties: the filtered DEGs (the comparison between acute and recovery) can be verified in the comparison between patients HC individuals; the filtered DEGs can eliminate the noise targets by the comparison between patients (recovery stage) and HC individuals; such comparisons allowed us to converge on targets with significant fold change (LogFC > 0.5). After filtering, we identified four genes, *CLU*, *PLCA8*, *CES1*, and *FTL* (Figure 1E), as potential exploitable transcriptomic biosignatures in HVA. To further confirm the potential roles of these selected DEGs in HVA, we performed correlation analysis to quantify the linear association in gene expression between each selected DEG and other common HVA DEGs in monocytes. Based on the results of the co‐expression analyses using the Pearson product‐moment correlation coefficient (PPMCC), we identified S100A8, S100A9, and S100A12 genes as co‐expressed genes of *CLU* gene in HVA patients, which also confirmed the roles of the selected DEGs based on the pathway analysis. These gene expression network may also support the roles of clusterin involved in the strong inflammatory processes^7^ or various stresses such as oxidative stress^8^ in human diseases.

To determine whether hypothetical developmental relationships exist between PBMCs, we performed single‐cell trajectory analysis to identify the pseudo‐temporal ordering of cells based on single‐cell transcriptomic datasets. A total of 13 marker genes (four HVA‐associated genes and nine *CLU*‐co‐expressed genes) were found to be involved in pseudo‐temporal ordering of cells into three different branches according to their characteristics. This pseudo‐temporal ordering was found to be applicable to defining the root nodes and direction of the trajectory (Figure S1). Notably, using pseudo‐temporal ordering analysis, CLU^+^ cells were classified into the same group as CES1^+^ cells and PLAC8^+^ cells, suggesting that the properties of disease‐associated cells can be identified from our feature selection strategies. To further investigate the dynamic changes in cell transitions and differentiation, we applied scVelo (a tool for analyzing RNA velocity analysis at the single‐cell level)^9^ to determine the direction of the monocyte cell population transition by evaluating the abundance of unspliced (nascent) and spliced (mature) mRNA. Analysis of RNA velocity information within the subset of three monocyte subsets (M1, M2, and M3 clusters) from all PBMCs in HVA patients indicated differentiation of M1 or M2 clusters toward the M3 cluster (Figure S2a). Moreover, analysis of RNA velocity information for three stages (acute, recovery, and HC) from all monocytes indicated the unique differentiation status of monocytes during the acute stage (Figure S2b). These findings supported that this differentiation of monocytes can be affected by stimulation with *Vespa* stings.

## 2 CONCLUSION

Our comprehensive molecular profiling of blood samples from patients with HVA revealed previously unknown molecular changes, providing important insights into mechanisms of venom allergy and potential therapeutic targets for precision medicine in HVA.

## FUNDING

We gratefully acknowledge the support of Ministry of Science and Technology (MOST‐107‐2314‐B‐009‐005‐MY2, MOST‐109‐2314‐B‐009‐003‐MY3), Center For Intelligent Drug Systems and Smart Bio‐devices (IDS2B) of National Yang Ming Chiao Tung University in Taiwan, National Health Research Institutes (NHRI‐EX111‐11140SI), and University System of Taiwan Joint Research Program (VGHUST108‐G2‐2‐1, VGHUST109‐V2‐1‐2) for their support.

## CONFLICT OF INTEREST

The authors declare no conflict of interest.

## AUTHOR CONTRIBUTIONS

Tai‐Ming Ko conceived the project and designed the experiments. Wen‐Ting Liao and Tai‐Ming Ko wrote the manuscript. Wen‐Cheng Chao recruited patients and provided clinical data. Wen‐Ting Liao, Jing‐Rong Wang, Hsiao‐Ni Sung, Fang‐Ping Lin, Jou‐Yu Huang, Kuan‐Ting Liu, and Tai‐Ming Ko designed and performed the bioinformatics analyses. Hsin‐Hua Chen and Wen‐Cheng Chao provided clinical comments. Jou‐Yu Huang and Hsiao‐Ni Sung contributed to the materials and experiments. Tai‐Ming Ko supervised the work.

## Supporting information

Supporting Information S1

Supporting Information S2

## Data Availability

The data that support the findings of this study are available on request from the corresponding author. The data are not publicly available due to privacy or ethical restrictions.
