# Supplementary material for: Single‐cell profiling reveals novel cellular heterogeneity of monocytes during Hymenoptera venom allergy
Source: Clin Transl Allergy. 2022 May 5;12(5):e12151. doi: 10.1002/clt2.12151 (PMC9069361; doi:10.1002/clt2.12151)
Supplement: Supplementary file 1 — Supporting Information S1 [file CLT2-12-e12151-s002.docx]

**Supporting information**

**Single-cell profiling reveals novel cellular heterogeneity of monocytes during Hymenoptera venom allergy**

**Wen-Cheng Chao^1#^, Wen-Ting Liao^2#^, Jing-Rong Wang^2^, Hsiao-Ni Sung^2^, Hsin-Hua Chen^3,4^, Fang-Ping Lin^2^, Jou-Yu Huang^2^, Kuan-Ting Liu^5^, Tai-Ming Ko^2,5,6,7*^**

^1^Department of Critical Care Medicine, Taichung Veterans General Hospital, Taichung, Taiwan.

^2^Department of Biological Science and Technology, National Yang Ming Chiao Tung University, Hsinchu, Taiwan.

^3^Division of Allergy, Immunology and Rheumatology, Department of Internal Medicine, Taichung Veterans General Hospital, Taichung, Taiwan.

^4^School of Medicine, National Yang Ming Chiao Tung University, Taipei, Taiwan.

^5^Institute of Bioinformatics and Systems Biology, National Chiao Tung University, Hsinchu, Taiwan.

^6^ Institute of Biomedical Sciences, Academia Sinica, Taipei, Taiwan.

^7^Center for Intelligent Drug Systems and Smart Bio-devices (IDS^2^B), National Yang Ming Chiao Tung University, Hsinchu, Taiwan.

**^#^These authors contributed equally to this work:** Wen-Cheng Chao, Wen-Ting Liao

***Correspondence should be addressed to:**

Tai-Ming Ko, PhD. ; Department of Biological Science and Technology, National Yang Ming Chiao Tung University; 75 Boai Street, Hsinchu 300, Taiwan; E-mail: [tmko@nctu.edu.tw](mailto:tmko@nctu.edu.tw); Phone: +886-3-571-2121#59731

**Running title: Single-cell profiling in Hymenoptera venom allergy**

**SUPPLEMENTARY METHODS**

**Ethical statement**

This investigation (CE19024B) was approved by the institutional review board and the ethics committee of the Taichung Veterans General Hospital, Taichung, Taiwan. Written informed consent was obtained from the subjects or their family members in accordance with the institutional requirements and Declaration of Helsinki principles.

**Patients**

Two patients had obvious symptoms after being attacked by the same *Vespa* group (*Vespa basalis*) in central Taiwan and were transferred to hospital within 8 hours. After plasma exchange, all patients recovered from hemolysis, rhabdomyolysis, and AKI. Patient_1 was a 44-year-old male who was conscious but not dyspnea, facial swelling or low blood pressure. Patient_2 was a 40-year-old woman with initially painful, dark red and itchy papules, conscious but severe breathing difficulties and severe acute respiratory distress syndrome (ARDS). Antihistamines and intravenous corticosteroids were given during the first treatment, and then plasma exchange was performed on the first day of hospitalization. They all had more than 50 stings with pustular skin lesions, and blood samples were taken from patients when they developed these skin lesions during this phase (the acute stage). After 14 days, these patients did not have any skin lesions, and their blood samples were also collected at this recovery stage.”

**TotalSeq-C antibody staining and 10x Chromium loading**

PBMCs samples (n=11) from two patients (acute stage and recovery stage) and seven healthy donors were separated using the SepMateTM. Cells exhibiting a viability rate less than 70% were excluded. Cells were quickly spun down at 400g, for 10 min. Viability was determined using trypan blue staining and measured on a Countess FLII. For labeling each sample for multiplexing, TotalSeqTM-C0251, TotalSeqTM-C0252, TotalSeqTM-C0253, TotalSeqTM-C0254, and TotalSeqTM-C0255 antibodies were used[^1^](#_ENREF_1). Samples were loaded onto a 10x Genomics microfluidics chip and encapsulated with barcoded hashtag-oligos (HTO)-containing gel beads using the 10x Genomics Chromium controller.

**Library preparation and sequencing**

Gene expression libraries were sequenced using a NovaSeq 6000 (Illumina) to achieve a minimum of 50,000 paired-end reads per cell. Cell-surface protein enrichment libraries were sequenced using a NovaSeq 6000 (Illumina) to achieve a minimum of 20,000 paired-end reads per cell.

**Analysis for scRNA-seq**

The output matrix of 10x Cell Ranger and CITE-Seq was processed with the R package Seurat v4.0.1, for visualization of the samples extracted from each reaction. The number of genes found in each cell (nFeature_ RNA) and the ratio of mitochondria (percent.mt) were used, which are the numerical values of the screening criterion. The threshold that gene numbers was greater than 4,000 but less than 200 and a mitochondrial percentage less than 5 was applied in this study. The NormalizeData (R package's function) process was applied to confirm that expression values across cells were on a comparable scale.

**Alignment and quantification for scRNA-seq**

The Cell Ranger Single Cell software (version 3.1.0.) were applied for matching raw reads to the human genome reference GRCh38. The cell-identifying barcodes, a unique molecular identifier (UMI), and a gene identifier for each read with a separate, strand-specific alignment to the exonic series were generated following the analysis pipeline (10x genomics). CITE-Seq-Count suite 1.4.3 (https://github.com/Hoohm/CITE-seq-Count) demultiplexed the gene-barcode matrix containing information on gene counts in each aggregated sample [^2^](#_ENREF_2)^,^[^3^](#_ENREF_3).

**Cluster differential abundance testing and annotation**

The modularity optimization techniques, such as the Louvain algorithm, were used to iteratively group cells together. For visualization, we integrated scRNA-seq data of PBMCs (5,836 cells) for unsupervised dimension-reduction clustering using uniform manifold approximation and projection (UMAP). SingleR (version 1.2.4), an automatic annotation method for single-cell RNA sequencing, was used to identify immune cell features and annotated clusters. Log fold-change of average expression (average logFC > 0.5) and Bonferroni-corrected (P <0.05) were the parameters. Popular DEGs of two patients were compared to seven healthy donors to investigate transcriptional profiles for each cell type and subpopulation[^4-6^](#_ENREF_4). The FindMarkers function from Seurat's package was applied for comparing expressed genes (DEGs) in clusters (or cell subpopulations) between patients and healthy donors.

**Single-cell trajectory analysis by Monocle**

Monocle v.2.8.0 was applied for analyzing inferred developmental trajectories of monocyte subsets. Differential expression analysis was performed using differentialGeneTest and the parameters HTO classification and the number of genes expressed. The reduceDimension command was used with the parameters max_ components=2 and reduction method=“DDRTree'' to minimize dimensionality. The plot cell trajectory command was then used with the default parameters to construct the trajectory. Cells were sorted into pseudotime order and placed along a trajectory based on differentially expressed genes that were discovered a priori. A heat map was created using a plot pseudotime heatmap to visualize changes in genes that are substantially branch-based[^7^](#_ENREF_7)^,^[^8^](#_ENREF_8).

**scRNA velocity analysis by scVelo**

To analyze the inferred velocity of cells, we used scVelo, a dynamic model, to compute cell dynamics and estimate RNA velocity of the cell transient state. This is a likelihood-based dynamical model that applies RNA velocity estimates to transient systems and systems with non-uniform subpopulation dynamics. First, scVelo's input data consists of two count matrices of pre-mature (unspliced) and mature (spliced) abundance. We utilize velocyto computation to extract RNA splicing information, and then these two count matrices are merged with the Seurat object. After the data uses filter_and_normalize for gene selection, and the normalized condition map, scv.pp.ments is used to calculate the mean and non-central variance. Then, scv. tl. velocity can be used to calculate the velocity and generate a cell dynamic map[^9^](#_ENREF_9).

**SUPPLEMENTARY FIGURE LEGENDS**

**Figure S1. Differentiation trajectory of monocytes in PBMCs from HVA patients**. (**a**) Monocle was used to order cells in pseudotime and allows the visualization of the differentiation process of different states in the monocytes clusters (left figure). (**b**) Expression heatmap of genes variable along the pseudotime trajectory (from Monocle) were analyzed in HVA patients (right figure). Each horizontal column represents one gene, and each vertical line represent one monocyte. Different colors correspond to the scaled (Z-scored) expression of each gene in each cell, from red (high) to blue (low).

**Figure S2. RNA dynamics revealed different spectrums of monocytes in HVA**. **(a)** RNA-velocity analysis of PBMCs clusters with velocity field projected onto the UMAP plot of PBMCs from patients with HVA (left figure). The arrows represent extrapolated future states of cells and the local average velocity evaluated on a regular grid (right figure). **(b)** RNA-velocity analysis of monocytes clusters with velocity field projected onto the UMAP plot of monocytes from patients with HVA (left figure). The arrows represent extrapolated future states of cells and the local average velocity evaluated on a regular grid (right figure).

**SUPPLEMENTARY REFERENCE**

1. Stoeckius M, Zheng S, Houck-Loomis B, et al. Cell Hashing with barcoded antibodies enables multiplexing and doublet detection for single cell genomics. *Genome Biol.* 2018;19(1):224.

2. Stoeckius M, Hafemeister C, Stephenson W, et al. Large-scale simultaneous measurement of epitopes and transcriptomes in single cells. *Nature methods.* 2017;14(9):865.

3. Stoeckius M, Hafemeister C, Stephenson W, et al. Simultaneous epitope and transcriptome measurement in single cells. *Nat Methods.* 2017;14(9):865-868.

4. Stuart T, Butler A, Hoffman P, et al. Comprehensive integration of single-cell data. *Cell.* 2019;177(7):1888-1902. e1821.

5. Satija R, Farrell JA, Gennert D, Schier AF, Regev A. Spatial reconstruction of single-cell gene expression data. *Nature biotechnology.* 2015;33(5):495-502.

6. Butler A, Hoffman P, Smibert P, Papalexi E, Satija R. Integrating single-cell transcriptomic data across different conditions, technologies, and species. *Nature biotechnology.* 2018;36(5):411-420.

7. Saelens W, Cannoodt R, Todorov H, Saeys Y. A comparison of single-cell trajectory inference methods. *Nature biotechnology.* 2019;37(5):547-554.

8. Bendall SC, Davis KL, Amir E-aD, et al. Single-cell trajectory detection uncovers progression and regulatory coordination in human B cell development. *Cell.* 2014;157(3):714-725.

9. Bergen V, Lange M, Peidli S, Wolf FA, Theis FJ. Generalizing RNA velocity to transient cell states through dynamical modeling. *Nature biotechnology.* 2020;38(12):1408-1414.
