# Supplementary material for: Single‐cell profiling reveals novel cellular heterogeneity of monocytes during Hymenoptera venom allergy
Source: Clin Transl Allergy. 2022 May 5;12(5):e12151. doi: 10.1002/clt2.12151 (PMC9069361; doi:10.1002/clt2.12151)
Supplement: Supplementary file 2 — Supporting Information S2 [file CLT2-12-e12151-s001.docx]

**SUPPLEMENTARY FIGURES**


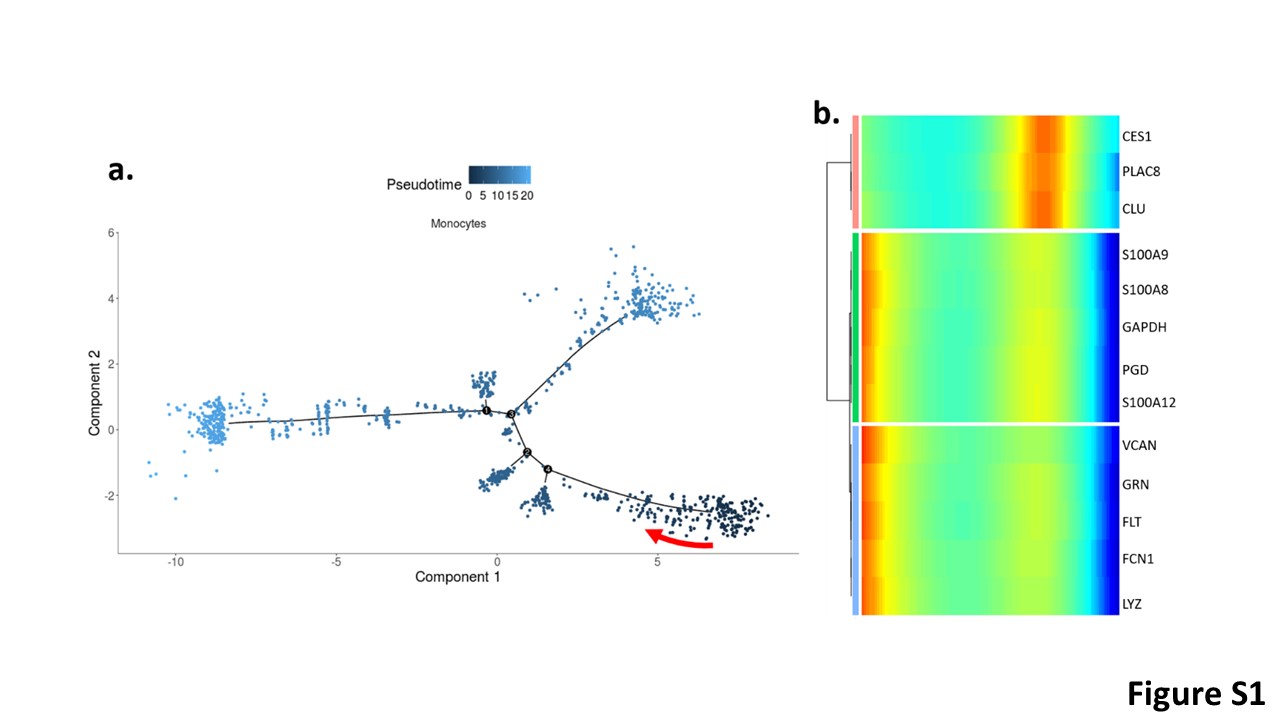


**Figure S1. Differentiation trajectory of monocytes in PBMCs from HVA patients**.


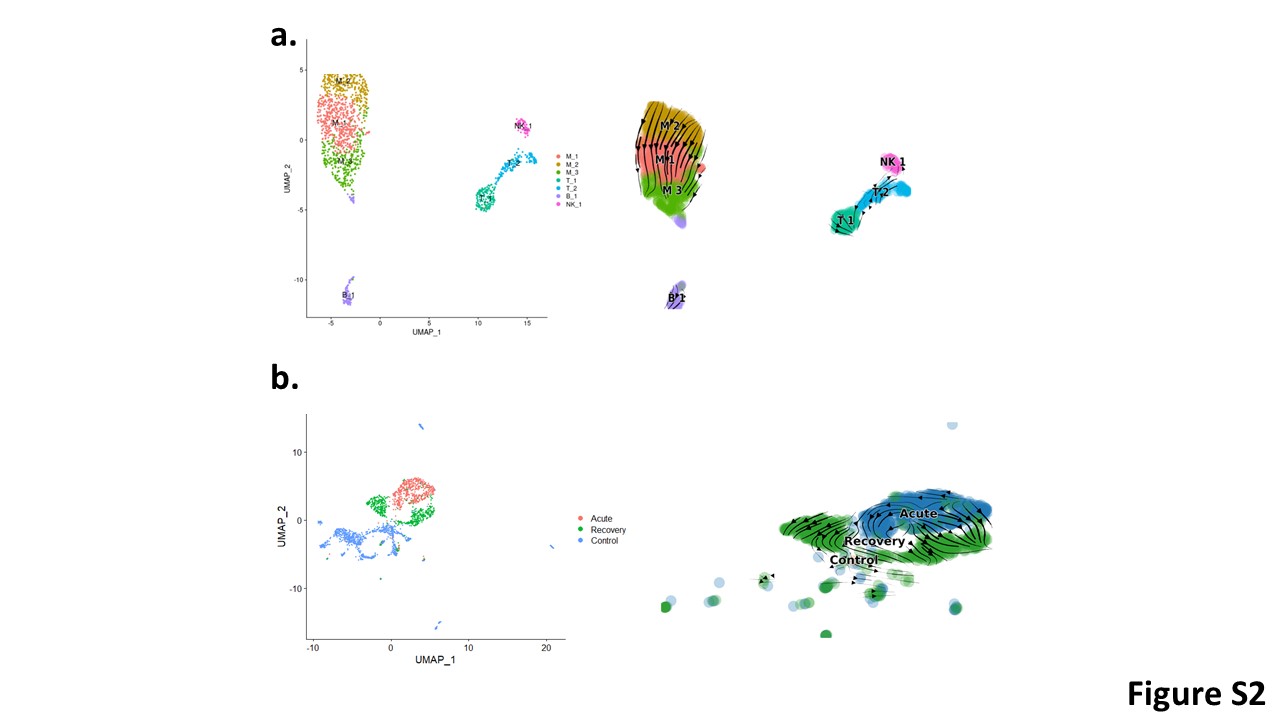


**Figure S2. RNA dynamics revealed different spectrums of monocytes in HVA**.
